# Bowman’s Practical Chemistry

**Published:** 1849-07

**Authors:** 


*Bowman's Practical Chemistry*—Philadelphia, 1849.

This is a duodecimo volume of some 300 pages, reprinted by Lea & Blanchard in their usual style of neatness and elegance. It is designed for those commencing the study of Practical Chemistry, and by the detail of manipulations, not usually given in treatises on analysis, to supply the wants of a numerous and increasing class of students. The book is divided into five parts. Part I. treats of the various manipulations which the Chemist has occasion to practice, and is illustrated abundantly with neat wood cuts. Part II. details the actions of reagents on acids and bases. Parts III. and IV. treat of qualitative or quantitative analysis. Part V. contains the examination of calculi, and an account of the reagents or substances employed in the processes for determining the character and composition of unknown bodies. Into an appendix are thrown certain tables, which are of constant use in the laboratory of the working chemist. We commend this book to beginners in the study of this most practical of all the sciences of this practical age. For sale by Derby & Co. G. H.

